# Predictors of sepsis in trauma patients: a National Trauma Data Bank analysis

**DOI:** 10.3389/fmed.2024.1500201

**Published:** 2024-12-20

**Authors:** Ralphe Bou Chebl, Joudie Sahar Alwan, Mounay Bakkar, Saadeddine Haidar, Rana Bachir, Mazen El Sayed, Gilbert Abou Dagher

**Affiliations:** Department of Emergency Medicine, American University of Beirut Medical Center, Beirut, Lebanon

**Keywords:** trauma, injury, Sepsis, risk factors, mortality

## Abstract

**Background:**

Trauma remains a global health issue being one of the leading causes of death worldwide. Sepsis and infections are common complications contributing to mortality, emphasizing the need to understand factors leading to such complications following trauma.

**Aim:**

This study aimed to identify risk factors associated with post-trauma sepsis using data from the National Trauma Data Bank (NTDB).

**Methods:**

Using the 2017 National Trauma Data Bank (NTDB), this is a retrospective case–control study that looked at pre-hospital and in-hospital patient data. Trauma patients aged over 15 years presenting to the emergency department (ED) and admitted to a tertiary care center were included. The primary outcome assessed was the development of sepsis post-trauma. Logistic regression analysis was used to identify risk factors, considering patient demographics, injury characteristics, and clinical variables.

**Results:**

Among 997,970 trauma patients in the 2017 NTDB, 296,974 were excluded, leaving 700,996 patients for analysis, with 2,297 developing sepsis. Patients who developed sepsis were older than those who did not develop sepsis (mean age 57.57 vs. 53.42 years, *p*-value<0.001) and predominantly white males. Risk factors associated with sepsis development included: respiratory intubation with mechanical ventilation (OR = 11.99; 95% CI = 10.66–13.48), blood transfusion administration (OR = 2.03; 95% CI = 1.83–2.25), Injury Severity Score (ISS) ≥ 16 (OR = 1.69; 95% CI = 1.51–1.89), chronic obstructive pulmonary disease (COPD) (OR = 1.65; 95% CI = 1.44–1.89), diabetes mellitus (DM) (OR = 1.41; 95% CI = 1.26–1.58), male sex (OR = 1.42; 95% CI = 1.28–1.57), hypertension (HTN) (OR = 1.30; 95% CI = 1.16–1.45), anticoagulation therapy (OR = 1.21; 95% CI = 1.05–1.39), older age (OR = 1.02; 95% CI = 1.01–1.02), and current smoking status (OR = 1.18; 95% CI = 1.06–1.32).

**Conclusion:**

This study identified key risk factors for post-trauma sepsis. Recognition of preexisting conditions and injury severity is crucial in trauma patient management to mitigate septic complications. Early identification of at-risk patients could facilitate timely interventions and potentially reduce mortality rates in trauma care settings.

## Introduction

Trauma accounts for approximately 6% of all deaths. It is the leading cause of death among those aged 1 to 44 years, and the fourth leading cause of mortality across all age groups (1). Sepsis and infections are potential complications of trauma due to alterations in the immune response, wound contaminations, invasive procedures, and surgeries (2). The development of sepsis after trauma has significant implications on healthcare cost, morbidity, and mortality (2–7). It is estimated that the median cost of hospitalization is nearly 3-fold higher in those who develop sepsis after trauma compared to those who did not develop sepsis after trauma, reaching an adjusted marginal cost of $16,646 (7). Also, it has been reported that patients who developed sepsis after trauma had significantly prolonged hospital/Intensive Care Unit (ICU) stay, higher rates of single or multi organ failure and are at increased risk of death when compared to those who did not develop sepsis after trauma (7). Thus, identifying trauma patients who are at higher risk of developing sepsis can help physicians in anticipating patient outcomes and initiate early interventions. Several factors associated with the development of sepsis post trauma have been previously studied and reported in the literature, including age, male sex, comorbidities, and severity of injuries (8–10). In this study, we aimed to identify risk factors associated with the development of sepsis post-trauma, using the largest trauma registry available, the NTDB.

## Methods

### Ethical approval and consent

This observational study was reviewed and approved by the Institutional Review Board (IRB) of the American University of Beirut (AUB). Given the retrospective nature of the study and the use of de-identified data from the NTDB, a waiver of informed consent was granted. The waiver was based on several factors: obtaining consent from all subjects would have been impractical and could introduce bias, many subjects were no longer actively followed, and comprehensive outcome data were necessary for statistical validity. Additionally, as the researchers were not involved in the clinical care of the subjects, no further ethical approval was required for this study. The NTDB data used in this research is fully anonymized, and the study was conducted under an umbrella IRB approval for all future research involving NTDB data, eliminating the need for reapplication.

### Study design

This study was a retrospective case control conducted by Emergency Medicine faculty members from the American University of Beirut in Lebanon. The 2017 National Trauma Data Bank (NTDB) was used. It is the largest trauma registry in the United States, and contains pre-hospital and in-hospital patient, injury and outcome related data (11).

### Inclusion/exclusion criteria

**Figure 1:**
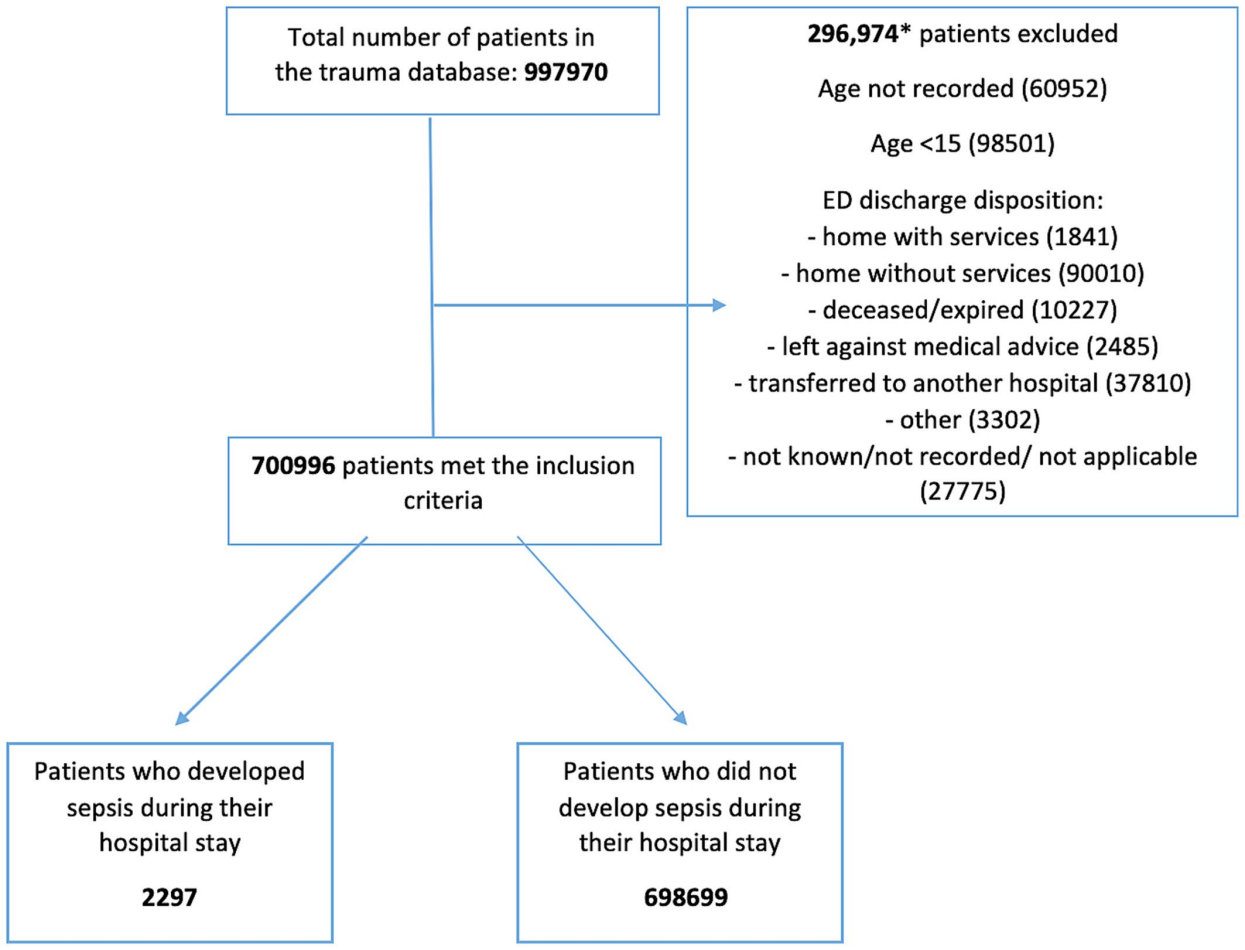
Flow diagram. This flow diagram illustrates the process for selecting and classifying patients from the database for the study. Stage 1 shows the total number of patients retrieved from the database. Stage 2 depicts the application of inclusion and exclusion criteria. In Stage 3, the patients who met the criteria are further categorized into two groups: those who developed sepsis and those who did not during their hospital stay. Arrows indicate the progression through each stage, and the numbers represent the count of patients at each stage of the process. The diagram highlights (specific points such as dropouts, exclusions, or key decisions) and provides a visual summary of the study methodology.

All trauma patients admitted to the hospital aged >15 years who developed sepsis as a complication were included. According to the NTDB, the diagnosis of sepsis must have been documented in the patient’s medical record and must have occurred during the patient’s initial stay at the hospital. The NTDB defines sepsis in accordance with the American College of Chest Physicians and the Society of Critical Care Medicine, which characterizes sepsis as life-threatening organ dysfunction caused by a dysregulated host response to infection. Patients ≤16 years or whose age was not known/not recorded were excluded. Those who were discharged from the emergency department (ED) home with/without services, died in the ED, left against medical advice, transferred to another hospital, discharged as “other” (jail, institutional care, mental health, etc.) and/or whose discharge disposition was not known/not recorded/ not applicable were excluded (Figure 1).

### Variables of interest

Data pertaining to the following variables were extracted from the NTDB: patient demographics, comorbidities at the time of their presentation [bleeding disorders, liver cirrhosis, Chronic Obstructive Pulmonary Disease (COPD), history of Cerebrovascular Accident (CVA), Diabetes Mellitus (DM), disseminated cancer, Congestive Heart Failure (CHF), Hypertension (HTN), history of Myocardial Infarction (MI), Peripheral Arterial Disease (PAD), and chronic renal failure, initial vital signs, injury related characteristics (location, nature, mechanism), therapeutic and/or diagnostic interventions (transfusion of blood or blood related products, surgeries, imaging), hospital complications and dispositions]. The primary outcome of interest was the development of sepsis post-trauma.

### Statistical analysis

The data analysis was performed using the Statistical Package for Social Sciences, version 25.0 (IBM, Armonk, New York, USA). The study sample was described by reporting frequencies and percentages for categorical variables, and calculating means, standard deviations, medians, and interquartile ranges (IQR) for continuous variables. The patients’ demographic and clinical characteristics were stratified using the hospital complication “sepsis” (yes/no). Pearson’s Chi-Square or the Fisher’s exact tests were then used to compare their percentages whereas the Student’s *t*-test was conducted to compare their means. To express accurate estimates, multiple imputation procedures were executed to account for the missing data (9.4%) of the variable “blood transfusion (4 h): Volume of packed red blood cells (RBCs) transfused (units or CCs) within first 4 h after ED/hospital arrival.” The conventional logistic regression is problematic if adopted as a multivariable approach in this study’s dataset because of the small number of patients who developed sepsis (2,297/997970 = 0.2%). To reduce the small-sample bias, the Firth logistic regression was conducted to estimate the odds ratios (ORs) along with the 95% confidence intervals (95% CIs) and to examine the association of hospital complication “sepsis” (yes/no) with the following independent factors: Age (years), Sex, Comorbidities [Anticoagulant therapy—COPD—DM—HTN—Current Smoker], Injury Severity Score (ISS), Glasgow Coma Scale (GCS), Systolic Blood Pressure (SBP), Pulse Rate, Respiratory Rate, Pulse Oximetry, Mechanism of Injury, Nature of injury, Procedures [Transfusion Blood—Computerized axial tomography (CT) scan—Respiratory intubation and mechanical ventilation—Routine chest X-ray —Suture of skin and subcutaneous tissue].

Variables’ selection was done to ensure that almost all patients’ demographic and clinical characteristics would be considered as potential associated factors with severe sepsis. To avoid ending up with a saturated model, the below measures were implemented while conducting the multivariable analysis: Only the top four most common comorbidities (COPD, DM, HTN, current smoker) were adjusted for. Moreover, only the top five most common procedures were adjusted for (transfusion blood, CT scan, Respiratory intubation and mechanical ventilation, routine chest X-ray, suture of skin and subcutaneous tissue). The trauma type was not included in the multivariable analysis because the majority of patients had blunt injuries, and because detailed information about it can be deducted from one of the considered confounding factors “mechanism of injury” and this can be achieved by relying on the CDC matrix that links each mechanism of injury to its corresponding trauma type and injury intentionality. Furthermore, some variables were not involved in the firth logistic regression (i.e., transfusion platelets, surgery for hemorrhage control type) due to their high percentages of missing values. A *p*-value of ≤0.05 was used to indicate statistical significance.

### Data availability

The data supporting the findings of this study are available from the corresponding author upon reasonable request. Requests for data access can be made directly to the corresponding author.

## Results

The total number of trauma patients in the 2,107 NTDB database was 997,970. 296,974 were excluded. Of the remaining 700,996 patients, 2,297 developed sepsis as a complication during their hospital stay and 698,699 did not (Figure 1).

The mean age of all patients was found to be 53 years with 60.8% of them being male and 73.8% of Caucasian race. The mean age of the population of patients who developed sepsis was higher than that of those who did not develop sepsis. More than half of the patients who developed sepsis post-trauma (59.1%) were older than 56 years of age. The majority of those who developed sepsis were also Caucasian males. When comparing those who developed sepsis to those who did not, septic patients were more likely to have chronic comorbidities such as a history of DM, COPD, stroke, and cancer (*p*-value <0.001 for DM, COPD, and stroke; *p*-value = 0.001 for cancer). Table 1 summarizes all the patient demographics and medical comorbidities.

**Table 1:** Demographics and medical comorbidities of presenting trauma patients.

	Total	Sepsis		*p*-value
	$N_{1}$ =700,996	No ( $N_{2}$ =698,699)	Yes ( $N_{3}$ =2,297)	
Age (years)	53.44 ± 21.73	53.42 ± 21.73	57.57 ± 19.45	<0.001
Sex				
Male	425,937 (60.8%)	424,273 (60.7%)	1,664 (72.5%)	<0.001
Female	274,970 (39.2%)	274,338 (39.3%)	632 (27.5%)	
Race^*^				
Black	96,459 (13.8%)	96,093 (14.0%)	366 (16.3%)	0.007
White	517,593 (73.8%)	515,956 (75.1%)	1,637 (72.9%)	
Other race	74,995 (10.7%)	74,751 (10.9%)	244 (10.9%)	
On anticoagulant therapy	56,197 (8.0%)	55,895 (8.0%)	302 (13.1%)	<0.001
Bleeding disorder	11,652 (1.7%)	11,579 (1.7%)	73 (3.2%)	<0.001
Currently receiving chemotherapy for cancer	3,040 (0.4%)	3,013 (0.4%)	27 (1.2%)	<0.001
Cirrhosis	8,772 (1.3%)	8,641 (1.2%)	131 (5.7%)	<0.001
Chronic obstructive pulmonary disease	50,037 (7.1%)	49,702 (7.1%)	335 (14.6%)	<0.001
Cerebrovascular accident	19,914 (2.8%)	19,801 (2.8%)	113 (4.9%)	<0.001
Diabetes mellitus	102,102 (14.6%)	101,567 (14.5%)	535 (23.3%)	<0.001
Disseminated cancer	4,537 (0.6%)	4,509 (0.6%)	28 (1.2%)	0.001
Congestive heart failure	27,450 (3.9%)	27,260 (3.9%)	190 (8.3%)	<0.001
Hypertension	245,251 (35.0%)	244,215 (35.0%)	1,036 (45.1%)	<0.001
Coronary artery disease	6,794 (1.0%)	6,754 (1.0%)	40 (1.7%)	<0.001
Peripheral artery disease	4,072 (0.6%)	4,039 (0.6%)	33 (1.4%)	<0.001
Chronic renal failure	12,474 (1.8%)	12,348 (1.8%)	126 (5.5%)	<0.001
Current smoker	151,436 (21.6%)	150,914 (21.6%)	522 (22.7%)	0.190
Steroid use	6,343 (0.9%)	6,297 (0.9%)	46 (2.0%)	<0.001

^*^Missing = 11,949 (1.7%). $N_{1}$ , total number of trauma patients. $N_{2}$ , total number of trauma patients who did not develop sepsis. $N_{3}$ , total number of trauma patients who developed sepsis. Out of 997,970 trauma patients in the 2017 NTDB database, 700,996 patients were included for analysis. Of these, 2,297 developed sepsis, with septic patients being older, predominantly male, and more likely to have chronic comorbidities like diabetes, COPD, and cancer compared to those who did not develop sepsis. Data obtained among subjects from the National Trauma Data Bank, 2024 United States of America.

Patients who developed sepsis had significantly lower systolic blood pressure at presentation (128 mmHg vs. 139 mmHg, *p*-value <0.001), higher pulse (96 bpm vs. 87 bpm, *p*-value <0.001), higher respiratory rate (20 vs. 18, *p*-value <0.001), and lower oxygen saturation on pulse oximetry (95% vs. 96%, *p*-value <0.001). Moreover, patients who developed sepsis had a lower GCS (GCS ≤ 8 21.4% vs. 5.9% and GCS 9–12 7.4% vs. 2.4%; *p*-value <0.001), as shown in the Supplementary Table S3.

Injury characteristics, in Supplementary Table S3, included ISS, trauma type, mechanism of injury, nature of injury and body region to which the injury was sustained. More than half of the patients who developed sepsis had a higher injury severity score upon presentation than those who did not develop sepsis (56.7% vs. 18.3% had an ISS ≥ 16, *p*-value <0.001). The most common trauma type in both patient groups was blunt trauma (84.2% in the sepsis group and 88.8% in the non-sepsis group, *p*-value <0.001). In terms of mechanism of injury, patients who developed sepsis were more likely to have been injured by firearm (9.4% vs. 4.5%, p-value <0.001) or by motor vehicle trauma (MVT) (33.8% vs. 28.8%, p-value <0.001). However, falls (42.6 and 46.4%) were the most common mechanism of injury among all patients presenting with trauma. The nature of injury among patients who did not develop sepsis was either a fracture (51.1%) or internal organ injury (24.8%). Among patients who developed sepsis post-trauma, 39.4% had fracture as the mechanism of injury and another 39.4% had internal organ injury with a *p*-value <0.001 making the nature of injury a significant risk factor for sepsis after trauma. The remaining nature of injury had similar distribution among both groups and all types of nature of injury were statistically significant. Body regions that were more commonly injured in patients who developed sepsis in contrast to those who did not include the spine, back (*p*-value = 0.038), and torso (*p*-value <0.001). Patients who did not develop sepsis were mainly injured at the extremities (*p*-value <0.001) or head and neck (*p*-value = 0.810).

Regarding treatment parameters, patients who developed sepsis received more blood and platelet transfusion, required intubation and mechanical ventilation, and underwent a greater number of surgeries such as laparotomies (6.1% vs. 0.5%; *p*-value <0.001) and chest and extremity related surgeries such as incision of pleura/thoracocentesis/chest drainage (31.8% vs. 5.5%, *p*-value <0.001), and laparoscopy (1.2% vs. 0.3%, *p*-value<0.001). All treatment related parameters are summarized in the Supplementary Table S4.

Admission to the operating room or the ICU was significantly higher in the sepsis group (25.7% vs. 13.1 and 48.1% vs. 23.2% respectively, *p*-value <0.001). These patients also had greater ICU length of stay (18.71 vs. 5.09 days, *p*-value <0.001) and total hospital length of stay (28.87 vs. 6.31 days, *p*-value <0.001). Total days spent on ventilation were also higher in the sepsis group (15.76 vs. 5.92 days, *p*-value <0.001). All dispositions and outcomes of trauma patients presenting to the ED are summarized in Supplementary Table S5.

Finally, firth logistic regression was performed on various risk factors to determine if they were statistically significant for sepsis development in presenting trauma patients. Male sex (OR = 1.42; 95% CI = 1.28–1.57), anticoagulation therapy (OR = 1.21; 95% CI = 1.05–1.39), COPD (OR = 1.65; 95% CI = 1.44–1.89), DM (OR = 1.41; 95% CI = 1.26–1.58), HTN (OR = 1.30; 95% CI = 1.16–1.45), current smoking status (OR = 1.18; 95% CI = 1.06–1.32), ISS ≥ 16 (OR = 1.69; 95% CI = 1.51–1.89), older age (OR = 1.02, 95% CI = 1.01–1.02), blood transfusion administration (OR = 2.03, 95% CI = 1.83–2.25), and respiratory intubation with mechanical ventilation (OR = 11.99, 95% CI = 10.66–13.48) were all found to be statistically significant risk factors for sepsis development (Table 2). Interestingly, the multivariable analysis also revealed that patients who developed sepsis post-trauma also had statistically significant higher ORs of all the comorbidities listed in Table 1 except for smoking status.

**Table 2:** Multivariable regression analysis showing the risk factors associated with sepsis in trauma patients.

Post-trauma sepsis risk factors	Adjusted odds ratio	95% CI	*p*-value
Age years	1.02	1.01–1.02	<0.001
Sex [Female]			
Male	1.42	1.28–1.57	<0.001
Race [Black]			
White	0.87	0.76–0.99	0.045
Other race	0.92	0.77–1.10	0.346
Anticoagulant therapy [No]			
Yes	1.21	1.05–1.39	0.009
Chronic obstructive pulmonary disease (COPD) [No]			
Yes	1.65	1.44–1.89	<0.001
Diabetes mellitus (DM) [No]			
Yes	1.41	1.26–1.58	<0.001
Hypertension (HTN) [No]			
Yes	1.30	1.16–1.45	<0.001
Current smoker [No]			
Yes	1.18	1.06–1.32	0.003
Mechanism of injury [Cut/pierce]			
Fall	1.64	1.18–2.28	0.002
Firearm	1.86	1.32–2.63	<0.001
MVT	1.50	1.09–2.08	0.010
Struck by, against	1.36	0.91–2.03	0.128
Other	1.76	1.24–2.49	0.001
ISS [≤15]			
≥16	1.69	1.51–1.89	<0.001
GCS [Mild 13–15]			
Moderate 9–12	0.91	0.76–1.10	0.329
Severe ≤8	0.66	0.58–0.75	<0.001
SBP [≤90]			
≥91	0.65	0.56–0.75	<0.001
Pulse rate	1.01	1.01–1.01	<0.001
Respiratory rate	1.01	1.00–1.02	0.008
Pulse oximetry	0.99	0.99–1.00	0.060
Nature of injury [Fracture]			
Internal organ injury	1.01	0.91–1.13	0.859
Open wound	0.92	0.76–1.12	0.390
Superficial and contusion	0.90	0.74–1.10	0.314
Other	1.22	1.02–1.45	0.031
Procedures [Blood transfusion] [No]			
Yes	2.03	1.83–2.25	<0.001
Procedures [Computerized axial tomography (CT) scan] [No]			
Yes	1.23	1.08–1.39	0.001
Procedures [Respiratory intubation and mechanical ventilation] [No]			
Yes	11.99	10.66–13.48	<0.001
Procedures [Routine chest X-ray] [No]			
Yes	1.23	1.12–1.36	<0.001
Procedures [Suture of skin and subcutaneous tissue] [No]			
Yes	1.03	0.92–1.16	0.605

Logistic regression identified several statistically significant risk factors for sepsis development in trauma patients, including male sex, certain comorbidities, older age, high injury severity, and the need for blood transfusions or mechanical ventilation. Additionally, almost all other comorbidities were also associated with increased odds of developing sepsis post-trauma. Data obtained among subjects from the National Trauma Data Bank, 2024 United States of America.

## Discussion

The results of our study showed that the incidence of sepsis post-trauma among adult patients was found to be 0.2%, which is lower than what has been described in the In the literature, with incidences ranging between 1 and 10% (8, 12, 13). Our study found that the mortality rate for trauma patients who develop sepsis is 33.7%, which is notably higher than the 20–23% mortality rates reported in previous studies (12, 13). By recognizing these patients early, timely interventions can be implemented, potentially reducing the risk of sepsis-related death.

Among trauma patients, older age and male sex were significantly associated with the development of post-trauma sepsis. Jin H et al. supported this by further explaining that older age is an independent risk factor since the elderly are more prone to developing sepsis due to a dysregulation of their physiological and immunological function (12–14). Furthermore, several studies have shown that males are at a higher risk of developing sepsis following trauma (8, 12, 13, 15). Some authors have stated that the lower association between female sex and mortality could be due to genetic and endocrine factors (15, 16). Raju et al. explained that estrogen plays an important role in the body’s response to sepsis as it not only stabilizes the immune reaction by decreasing cytokine release but also has a favorable effect on the cardiovascular system (17, 18).

Preexisting medical conditions have been previously linked to sepsis following traumatic injury (8, 13, 19). Our study showed that preexisting medical conditions such as DM, HTN, COPD, smoking status, and the use of anticoagulants were independent risk factors that predispose to the development of sepsis post-trauma. In the literature, one study showed through logistic regression that diabetes mellitus was found to be one of the pre-existing comorbid conditions that is an independent risk factor for sepsis development post-injury (13). Diabetes not only increases the risk of sepsis with an OR of 1.61 and a statistically significant *p* value of 0.02, but it also demonstrated a correlation with longer hospital stay and ICU admission (13). Furthermore, COPD and smoking are known to be independent risk factors for sepsis and increased mortality in patients hospitalized with sepsis due to pneumonia (20, 21). It is well documented that both smoking and COPD impair the structural and immunological defense mechanisms of the body (20, 21). Smoking causes peribronchiolar inflammation and fibrosis which results in an alteration in mucosal permeability and deterioration in the function of the mucociliary clearance therefore increasing susceptibility to infection (20, 22). Similarly, COPD is associated with chronic airway inflammation and impaired immune responses, further predisposing individuals to respiratory infections (21). Trauma can lead to sepsis through complex immune responses that typically begin with the body’s natural defense to injury, which can become dysregulated. After trauma, many pro-inflammatory cytokines, such as TNF-*α*, IL-1 and IL-6 are released as well as anti-inflammatory cytokines, such as IL-10. The release of these cytokines leads to immune suppression, which increases susceptibility to secondary infections, ultimately raising the risk of sepsis and its complications (14). Additionally, numerous chronic diseases, such as cancer, diabetes and CAD, are linked to chronic inflammation, which may further increase the risk of progression to sepsis when exposed to certain pathogens (23).

The use of anticoagulation as well as blood transfusions were found to be risk factors for sepsis as well. Anticoagulant therapy is indeed considered a comorbidity in the NTDB because it may result in severe bleeding, complicating trauma management and can worsen outcomes (24). To the best of our knowledge, there are no studies in the literature that have identified anticoagulation use as a risk factor for sepsis development post trauma. On the other hand, studies on the epidemiology of sepsis in traumatic injury patient have shown that blood transfusions are indeed independent risk factors for sepsis development (13). One possible explanation is that anticoagulation use might predispose trauma patients to require more blood transfusion and might increase their length of stay in the hospital which might put them at an increased risk of sepsis. Furthermore, blood transfusions are believed to harbor inflammatory mediators within packed red blood cells (RBCs), that can potentially trigger a systemic inflammatory response as well as induce widespread immunosuppression (8, 25–29).

Finally, this study demonstrated that the ISS and the need for intubation were predictors of sepsis development. Papia et al. previously described that requirement for intubation and mechanical ventilation were associated with an increased risk for infection and correlated with greater severity of illness (26). The increased risk of sepsis could be due to the intubation process itself, which can facilitate direct pathogen entry into the airway tract, bypassing natural defense mechanisms. Moreover, prolonged tube usage contributes to damage of the tracheal ciliated epithelium, impairing the effective clearance of bacteria (30, 31). Numerous previous articles proved that patients who developed sepsis post-trauma had higher ISS and lower GCS (2, 8, 12–14, 25, 26). In contrast, our results indicated that a lower GCS score is associated with a reduced risk of developing sepsis following trauma. GCS is a score that denotes the physiological impairment caused by trauma (14). Lower GCS scores may not only reflect compromised neurological function but also suggest a deeper level of physiological stress and immune dysregulation rendering patients more susceptible to sepsis (14, 32, 33). Although this finding contrasts with previous results, a GCS score of less than 8 showed to be protective in our study. This could potentially be because such patients are often more severely ill and receive more intensive care compared to their more stable counterparts, necessitating higher levels of care.

Several limitations must be considered for this study. One limitation of using a large dataset is that even minor differences between factors can become statistically significant, which might not be observed in smaller studies. Despite this, the substantial sample size of our dataset provides robust and generalizable insights into the predictors of sepsis, enhancing the reliability of our findings. Additionally, this study did not include any laboratory test results such as microbiology findings making it unclear whether some patients had infections that might have contributed to their condition, whether or not those infections developed into sepsis. This lack of data limits the ability to fully understand the role infections may have played in the development of sepsis or other health outcomes. The retrospective nature of this study poses another drawback. The NTDB is a retrospective dataset, and underreporting of complications is known to be common. In addition, the database mostly includes hospitals located in the United States, which can limit the generalizability of this study to other populations. There might be differences in the data quality collected from the different hospitals in the NTDB. Furthermore, the NTDB does not offer information on the classic predictors of sepsis such as lactate, procalcitonin and SOFA score. Despite its limitations, the database’s large sample size enables robust research outcomes and supports the study of clinical outcomes across multiple institutions, offering valuable insights into injury quantification and trauma care quality (34).

## Conclusion

This retrospective study utilized data extracted from the NTDB to investigate potential predictors of sepsis development in trauma patients. Our analysis identified male sex, advanced age, and comorbidities such as COPD, smoking, diabetes mellitus, hypertension, and anticoagulant therapy as significant risk factors for sepsis development in trauma patients. Furthermore, patients with ISS ≥ 16, and individuals needing intubations and blood transfusions were associated with an increased risk of sepsis. Determining those risk factors can help physicians quickly identify trauma patients at risk of developing sepsis. By incorporating these findings into clinical practice, healthcare providers can better prioritize early screening, monitoring, and interventions for high-risk patients, potentially improving patient outcomes through more timely and targeted care pathways.

## Data Availability

The raw data supporting the conclusions of this article will be made available by the authors, without undue reservation.

## References

[1] Maerz LL Davis KA Rosenbaum SH. Trauma. Int Anesthesiol Clin. (2009) 47:25–36. doi: 10.1097/AIA.0b013e3181950030 19131750

[2] Brattström O Granath F Rossi P Oldner A. Early predictors of morbidity and mortality in trauma patients treated in the intensive care unit. Acta Anaesthesiol Scand. (2010) 54:1007–17. doi: 10.1111/j.1399-6576.2010.02266.x, PMID: 20626360

[3] Plurad DS Lustenberger T Kilday P Zhu J Green DJ Inaba K. The association of race and survival from sepsis after injury. Am Surg. (2010) 76:43–7. doi: 10.1177/000313481007600109, PMID: 20135938

[4] Fitzwater J Purdue GF Hunt JL O'Keefe GE. The risk factors and time course of sepsis and organ dysfunction after burn trauma. J Trauma. (2003) 54:959–66. doi: 10.1097/01.TA.0000029382.26295.AB, PMID: 12777910

[5] Muckart DJ Bhagwanjee S. American College of Chest Physicians/Society of Critical Care Medicine consensus conference definitions of the systemic inflammatory response syndrome and allied disorders in relation to critically injured patients. Crit Care Med. (1997) 25:1789–95. doi: 10.1097/00003246-199711000-00014, PMID: 9366759

[6] Glance LG Stone PW Mukamel DB Dick AW. Increases in mortality, length of stay, and cost associated with hospital-acquired infections in trauma patients. Arch Surg. (2011) 146:794–801. doi: 10.1001/archsurg.2011.41, PMID: 21422331 PMC3336161

[7] Eguia E Bunn C Kulshrestha S Markossian T Durazo-Arvizu R Baker MS. Trends, cost, and mortality from Sepsis after trauma in the United States: an evaluation of the National Inpatient Sample of hospitalizations, 2012-2016. Crit Care Med. (2020) 48:1296–303. doi: 10.1097/CCM.0000000000004451, PMID: 32590387 PMC7872079

[8] Wafaisade A Lefering R Bouillon B Sakka SG Thamm OC Paffrath T. Epidemiology and risk factors of sepsis after multiple trauma: an analysis of 29,829 patients from the trauma registry of the German Society for Trauma Surgery. Crit Care Med. (2011) 39:621–8. doi: 10.1097/CCM.0b013e318206d3df, PMID: 21242798

[9] Eguia E Cobb AN Baker MS Joyce C Gilbert E Gonzalez R. Risk factors for infection and evaluation of Sepsis-3 in patients with trauma. Am J Surg. (2019) 218:851–7. doi: 10.1016/j.amjsurg.2019.03.005, PMID: 30885453 PMC6732249

[10] Lakomkin N Sathiyakumar V Wick B Shen MS Jahangir AA Mir H. Incidence and predictive risk factors of postoperative sepsis in orthopedic trauma patients. J Orthop Traumatol. (2017) 18:151–8. doi: 10.1007/s10195-016-0437-4, PMID: 27848054 PMC5429254

[11] Hashmi ZG Kaji AH Nathens AB. Practical guide to surgical data sets: National Trauma Data Bank (NTDB). JAMA Surg. (2018) 153:852–3. doi: 10.1001/jamasurg.2018.0483, PMID: 29617536

[12] Kisat M Villegas CV Onguti S Zafar SN Latif A Efron DT. Predictors of sepsis in moderately severely injured patients: an analysis of the National Trauma Data Bank. Surg Infect. (2013) 14:62–8. doi: 10.1089/sur.2012.009, PMID: 23461696 PMC3601717

[13] Osborn TM Tracy JK Dunne JR Pasquale M Napolitano LM. Epidemiology of sepsis in patients with traumatic injury. Crit Care Med. (2004) 32:2234–40. doi: 10.1097/01.CCM.0000145586.23276.0F, PMID: 15640635

[14] Jin H Liu Z Xiao Y Fan X Yan J Liang H. Prediction of sepsis in trauma patients. Burns Trauma. (2014) 2:106–13. doi: 10.4103/2321-3868.135479, PMID: 27602370 PMC5012019

[15] Oberholzer A Keel M Zellweger R Steckholzer U Trentz O Ertel W. Incidence of septic complications and multiple organ failure in severely injured patients is sex specific. J Trauma. (2000) 48:932–7. doi: 10.1097/00005373-200005000-00019, PMID: 10823539

[16] Haider AH Crompton JG Chang DC Efron DT Haut ER Handly N. Evidence of hormonal basis for improved survival among females with trauma-associated shock: an analysis of the National Trauma Data Bank. J Trauma. (2010) 69:537–40. doi: 10.1097/TA.0b013e3181efc67b, PMID: 20838123

[17] Knöferl MW Angele MK Diodato MD Schwacha MG Ayala A Cioffi WG. Female sex hormones regulate macrophage function after trauma-hemorrhage and prevent increased death rate from subsequent sepsis. Ann Surg. (2002) 235:105–12. doi: 10.1097/00000658-200201000-00014, PMID: 11753049 PMC1422402

[18] Raju R Chaudry IH. Sex steroids/receptor antagonist: their use as adjuncts after trauma-hemorrhage for improving immune/cardiovascular responses and for decreasing mortality from subsequent sepsis. Anesth Analg. (2008) 107:159–66. doi: 10.1213/ane.0b013e318163213d, PMID: 18635483

[19] Gannon CJ Napolitano LM Pasquale M Tracy JK McCarter RJ. A statewide population-based study of gender differences in trauma: validation of a prior single-institution study. J Am Coll Surg. (2002) 195:11–8. doi: 10.1016/S1072-7515(02)01187-0, PMID: 12113534

[20] Alroumi F Abdul Azim A Kergo R Lei Y Dargin J. The impact of smoking on patient outcomes in severe sepsis and septic shock. J Intensive Care. (2018) 6:42. doi: 10.1186/s40560-018-0312-x, PMID: 30065844 PMC6064183

[21] Inghammar M Engström G Ljungberg B Löfdahl CG Roth A Egesten A. Increased incidence of invasive bacterial disease in chronic obstructive pulmonary disease compared to the general population--a population based cohort study. BMC Infect Dis. (2014) 14:163. doi: 10.1186/1471-2334-14-163, PMID: 24661335 PMC3976148

[22] Arcavi L Benowitz NL. Cigarette smoking and infection. Arch Intern Med. (2004) 164:2206–16. doi: 10.1001/archinte.164.20.2206 15534156

[23] Wang HE Shapiro NI Griffin R Safford MM Judd S Howard G. Chronic medical conditions and risk of sepsis. PLoS One. (2012) 7:e48307. doi: 10.1371/journal.pone.0048307, PMID: 23118977 PMC3485139

[24] Nguyen RK Rizor JH Damiani MP Powers AJ Fagnani JT Monie DL. The impact of anticoagulation on trauma outcomes: an National Trauma Data Bank Study. Am Surg. (2020) 86:773–81. doi: 10.1177/0003134820934419, PMID: 32730098

[25] Croce MA Fabian TC Waddle-Smith L Maxwell RA. Identification of early predictors for post-traumatic pneumonia. Am Surg. (2001) 67:105–10. doi: 10.1177/000313480106700201, PMID: 11243529

[26] Papia G McLellan BA El-Helou P Louie M Rachlis A Szalai JP. Infection in hospitalized trauma patients: incidence, risk factors, and complications. J Trauma. (1999) 47:923–7. doi: 10.1097/00005373-199911000-00018 10568723

[27] Moore FA Moore EE Sauaia A. Blood transfusion. An independent risk factor for postinjury multiple organ failure. Arch Surg. (1997) 132:620–4. doi: 10.1001/archsurg.1997.01430300062013 9197854

[28] Shalhub S Junker CE Imahara SD Mindrinos MN Dissanaike S O'Keefe GE. Variation in the TLR4 gene influences the risk of organ failure and shock posttrauma: a cohort study. J Trauma. (2009) 66:115–22. doi: 10.1097/TA.0b013e3181938d50 19131814 PMC2740632

[29] Bochicchio GV Napolitano L Joshi M Bochicchio K Meyer W Scalea TM. Outcome analysis of blood product transfusion in trauma patients: a prospective, risk-adjusted study. World J Surg. (2008) 32:2185–9. doi: 10.1007/s00268-008-9655-0, PMID: 18575931

[30] Tejada Artigas A Bello Dronda S Chacón Vallés E Muñoz Marco J Villuendas Usón MC Figueras P. Risk factors for nosocomial pneumonia in critically ill trauma patients. Crit Care Med. (2001) 29:304–9. doi: 10.1097/00003246-200102000-00015, PMID: 11246310

[31] Baker AM Meredith JW Haponik EF. Pneumonia in intubated trauma patients. Microbiology and outcomes. Am J Respir Crit Care Med. (1996) 153:343–9. doi: 10.1164/ajrccm.153.1.8542141, PMID: 8542141

[32] Mas-Celis F Olea-López J Parroquin-Maldonado JA. Sepsis in trauma: a deadly complication. Arch Med Res. (2021) 52:808–16. doi: 10.1016/j.arcmed.2021.10.007, PMID: 34706851

[33] Middleton PM. Practical use of the Glasgow coma scale; a comprehensive narrative review of GCS methodology. Australas Emerg Nurs J. (2012) 15:170–83. doi: 10.1016/j.aenj.2012.06.002, PMID: 22947690

[34] Haider AH Saleem T Leow JJ Villegas CV Kisat M Schneider EB. Influence of the National Trauma Data Bank on the study of trauma outcomes: is it time to set research best practices to further enhance its impact? J Am Coll Surg. (2012) 214:756–68. doi: 10.1016/j.jamcollsurg.2011.12.013, PMID: 22321521 PMC3334459

