# Supplementary material for: Predictors of sepsis in trauma patients: a National Trauma Data Bank analysis
Source: Front Med (Lausanne). 2024 Dec 20;11:1500201. doi: 10.3389/fmed.2024.1500201 (PMC11697700; doi:10.3389/fmed.2024.1500201)
Supplement: Supplementary file 1 [file Table_1.DOCX]

|  | **Total** | **Sepsis** | | **p-value** |
| --- | --- | --- | --- | --- |
|  | $\boldsymbol{N}_{\boldsymbol{1}}$**=700996** | **No**  **(**$\boldsymbol{N}_{\boldsymbol{2}}$**=698699)** | **Yes**  **(**$\boldsymbol{N}_{\boldsymbol{3}}$**=2297)** |  |
| **SBP (mmHg)**  ≤ 90  ≥ 91 | 18855 (2.7%)  667841 (95.3%) | 18566 (2.7%)  665890 (97.3%) | 289 (12.9%)  1951 (87.1%) | <0.001 |
| **SBP (mmHg)** | 139.55 ± 27.1 | 139.59 ± 27.07 | 128.51 ± 34.33 | <0.001 |
| **Pulse Rate (beats/minute)** | 87.42 ± 19.6 | 87.39 ± 19.58 | 96.9 ± 24.87 | <0.001 |
| **Respiratory Rate (breaths/minute)** | 18.49 ± 4.41 | 18.49 ± 4.4 | 20.01 ± 6.44 | <0.001 |
| **Pulse Oximetry (%)** | 96.92 ± 4.86 | 96.92 ± 4.85 | 95.67 ± 7.48 | <0.001 |
| **GCS**  Severe ≤ 8  Moderate 9 – 12  Mild 13 – 15 | 39493 (5.6%)  16417 (2.3%)  612770 (87.4%) | 39025 (5.9%)  16255 (2.4%)  611211 (91.7%) | 468 (21.4%)  162 (7.4%)  1559 (71.2%) | <0.001  <0.001  <0.001 |
| **ISS**  ≤ 15  ≥ 16 | 570500 (81.4%)  129077 (18.4%) | 569506 (81.7%)  127777 (18.3%) | 994 (43.3%)  1300 (56.7%) | <0.001 |
| **Trauma type**  Blunt  Penetrating  Burn  Unspecified | 617877 (88.1%)  62902 (9.0%)  6798 (1.0%)  8513 (1.2%) | 615961 (88.8%)  62636 (9.0%)  6743 (1.0%)  8475 (1.2%) | 1916 (84.2%)  266 (11.7%)  55 (2.4%)  38 (1.7%) | <0.001  <0.001  <0.001  0.052 |
| **Mechanism of Injury**  Cut/pierce  Fall  Firearm  Motor vehicle transport  Struck by, against  Other* | 29043 (4.1%)  321309 (45.8%)  31320 (4.5%)  199625 (28.5%)  43891 (6.3%)  67557 (9.6%) | 28995 (4.2%)  320343 (46.4%)  31106 (4.5%)  198857 (28.8%)  43815 (6.3%)  67359 (9.8%) | 48 (2.1%)  966 (42.6%)  214 (9.4%)  768 (33.8%)  76 (3.3%)  198 (8.7%) | <0.001  <0.001  <0.001  <0.001  <0.001  0.098 |
| **Nature of injury**  Fracture  Internal organ injury  Open wound  Superficial and contusion  Other^‡^ | 356646 (50.9%)  173303 (24.7%)  68401 (9.8%)  48221 (6.9%)  51918 (7.4%) | 355745 (51.1%)  172402 (24.8%)  68251 (9.8%)  48092 (6.9%)  51715 (7.4%) | 901 (39.4%)  901 (39.4%)  150 (6.6%)  129 (5.6%)  203 (8.9%) | <0.001  <0.001  <0.001  0.018  0.008 |

* Other mechanism of injury: Drowning/submersion & Fire/flame & Hot object/substance & Machinery & Pedal cyclist, other & Pedestrian, other & Transport, other & Natural/environmental, Bites and stings & Natural/environmental, Other & Overexertion & Poisoning & Suffocation & Other specified and classifiable & Other specified, not elsewhere classifiable & Unspecified

^‡^ Other nature of injury: Amputation & Blood vessel & burns and corrosions & Crushing & Dislocation&Effect of foreign body entering orifice& Other effects of external causes & Other specified injury &Poisoning & Toxic effects &Unspecified injury

$\boldsymbol{N}_{\boldsymbol{1}}$**= total number of trauma patients**

$\boldsymbol{N}_{\boldsymbol{2}}$**= total number of trauma patients who didn’t develop sepsis**

$\boldsymbol{N}_{\boldsymbol{3}}$**= total number of trauma patients who developed sepsis**

**Table 3:** Variables at presentation of trauma patients

Patients who developed sepsis had lower systolic blood pressure, higher pulse, respiratory rate, and lower oxygen saturation compared to those who did not. They also had lower GCS scores, higher injury severity scores, and were more likely to suffer from firearm or motor vehicle trauma, and injuries to the spine, back, or torso, with blunt trauma and falls being the most common for both.

*Data obtained among subjects from the National Trauma Data Bank, 2024*

*United States of America*

| **Procedures** | **Total** | **Sepsis** | | **p-value** |
| --- | --- | --- | --- | --- |
|  | $\boldsymbol{N}_{\boldsymbol{1}}$**= 700996** | **No**  **(**$\boldsymbol{N}_{\boldsymbol{2}}$**=698699)** | **Yes**  **(**$\boldsymbol{N}_{\boldsymbol{3}}$**=2297)** |  |
| **Transfusion blood (4 hours)** | 95497 (13.6%) | 94797 (13.6%) | 700 (30.5%) | <0.001 |
| **Transfusion Platelets (4 Hours)** | 10123 (1.4%) | 9841 (33.8%) | 282 (48.9%) | <0.001 |
| **Transfusion Platelets (24 Hours)** | 11876 (1.7%) | 11534 (39.6%) | 342 (59.3%) | <0.001 |
| **Transfusion Blood (4 Hours) (mL)** | 245.44 ± 719.7 | 244.66 ± 715.95 | 351.37 ± 1112.73 | 0.012 |
| **Transfusion Blood (24 Hours) (mL)** | 361.15 ± 1593.22 | 356.80 ± 1582.4 | 579.89 ± 2055.26 | 0.009 |
| **Cystoscopy and other transurethral procedures** | 4838 (0.7%) | 4748 (0.7%) | 90 (3.9%) | <0.001 |
| **Exploratory laparotomy** | 3732 (0.5%) | 3593 (0.5%) | 139 (6.1%) | <0.001 |
| **Hemodialysis** | 3290 (0.5%) | 3060 (0.4%) | 230 (10.0%) | <0.001 |
| **Incision of pleura; thoracentesis; chest drainage** | 39096 (5.6%) | 38366 (5.5%) | 730 (31.8%) | <0.001 |
| **Indwelling catheter** | 53456 (7.6%) | 52880 (7.6%) | 576 (25.1%) | <0.001 |
| **Laparoscopy** | 2343 (0.3%) | 2315 (0.3%) | 28 (1.2%) | <0.001 |
| **Respiratory intubation and mechanical ventilation** | 72759 (10.4%) | 71204 (10.2%) | 1555 (67.7%) | <0.001 |

$\boldsymbol{N}_{\boldsymbol{1}}$**= total number of trauma patients**

$\boldsymbol{N}_{\boldsymbol{2}}$**= total number of trauma patients who didn’t develop sepsis**

$\boldsymbol{N}_{\boldsymbol{3}}$**= total number of trauma patients who developed sepsis**

**Table 4:** Procedures underwent by the presenting trauma patients.

Patients who developed sepsis required more intensive treatments, including blood and platelet transfusions, intubation and mechanical ventilation, and a higher number of surgeries (laparotomies, chest-related surgeries, laparoscopy) compared to those who didn’t develop sepsis.

*Data obtained among subjects from the National Trauma Data Bank, 2024*

*United States of America*

|  | **Total** | **Sepsis** | | **p-value** |
| --- | --- | --- | --- | --- |
|  | $\boldsymbol{N}_{\boldsymbol{1}}$**= 700996** | **No**  **(**$\boldsymbol{N}_{\boldsymbol{2}}$**=698699)** | **Yes**  **(**$\boldsymbol{N}_{\boldsymbol{3}}$**=2297)** |  |
| **ED Discharge Disposition** |  |  |  |  |
| General floor bed | 344022 (49.1%) | 343603 (49.2%) | 419 (18.2%) | <0.001  <0.001  <0.001  <0.001  <0.001 |
| Observation unit (<24-hour stay) | 29317 (4.2%) | 29290 (4.2%) | 27 (1.2%) |  |
| Telemetry/step-down unit (less acuity than ICU) | 72270 (10.3%) | 72115 (10.3%) | 155 (6.7%) |  |
| Operating Room | 92348 (13.2%) | 91758 (13.1%) | 590 (25.7%) |  |
| Intensive Care Unit (ICU) | 163039 (23.3%) | 161933 (23.2%) | 1106 (48.1%) |  |
| **Hospital Discharge Disposition**  Deceased/Expired  Left against medical advice  Discharged to home  Transferred to other destination  Not Applicable | 22207 (3.2%)  9036 (1.3%)  394880 (56.3%)  274722 (39.2%)  151 (0%) | 21434 (3.1%)  9021 (1.3%)  394660 (56.5%)  273435 (39.1%)  149 (0%) | 773 (33.7%)  15 (0.7%)  220 (9.6%)  1287 (56.0%)  2 (0.1%) | <0.001  0.007  <0.001  <0.001  0.088* |
| **Length of stay (days)** | 6.39 ± 8.8 | 6.31 ± 8.59 | 28.87 ± 27.45 | <0.001 |
| **Total ICU Length of Stay (days)** | 5.23 ± 6.84 | 5.09 ± 6.52 | 18.71 ± 17.24 | <0.001 |
| **Total Vent Days** | 6.14 ± 8.35 | 5.92 ± 7.98 | 15.76 ± 15.6 | <0.001 |

$\boldsymbol{N}_{\boldsymbol{1}}$**= total number of trauma patients**

$\boldsymbol{N}_{\boldsymbol{2}}$**= total number of trauma patients who didn’t develop sepsis**

$\boldsymbol{N}_{\boldsymbol{3}}$**= total number of trauma patients who developed sepsis**

**Table 5:** Disposition and outcomes of presenting trauma patients

Patients who developed sepsis had higher rates of operating room and ICU admissions, longer total hospital and ICU stay, with more ventilation support compared to those without sepsis.

*Data obtained among subjects from the National Trauma Data Bank, 2024*

*United States of America*
